# Complexes of Cationic Pyridylphenylene Dendrimers with Anionic Liposomes: The Role of Dendrimer Composition in Membrane Structural Changes

**DOI:** 10.3390/ijms24032225

**Published:** 2023-01-22

**Authors:** Anna A. Efimova, Svetlana A. Sorokina, Kseniya S. Trosheva, Alexander A. Yaroslavov, Zinaida B. Shifrina

**Affiliations:** 1 Department of Chemistry, M.V. Lomonosov Moscow State University, 1-3 Leninskie Gory, 119991 Moscow, Russia; 2 A.N. Nesmeyanov Institute of Organoelement Compounds, Russian Academy of Sciences, 28 Vavilov St., 119991 Moscow, Russia

**Keywords:** dendrimer, liposome, biomembrane, complex, lipid bilayer, migration

## Abstract

In the last decades, dendrimers have received attention in biomedicine that requires detailed study on the mechanism of their interaction with cell membranes. In this article, we report on the role of dendrimer structure in their interaction with liposomes. Here, the interactions between cationic pyridylphenylene dendrimers of the first, second, and third generations with mixed or completely charged pyridyl periphery (D_1_^6+^, D_2_^15+^, D_2_^29+^, and D_3_^50+^) with cholesterol-containing (CL/Chol/DOPC) anionic liposomes were investigated by microelectrophoresis, dynamic light scattering, fluorescence spectroscopy, and conductometry. It was found that the architecture of the dendrimer, namely the generation, the amount of charged pyridynium groups, the hydrophobic phenylene units, and the rigidity of the spatial structure, determined the special features of the dendrimer–liposome interactions. The binding of D_3_^50+^ and D_2_^29+^ with almost fully charged peripheries to liposomes was due to electrostatic forces: the dendrimer molecules could be removed from the liposomal surfaces by NaCl addition. D_3_^50+^ and D_2_^29+^ did not display a disruptive effect toward membranes, did not penetrate into the hydrophobic lipid bilayer, and were able to migrate between liposomes. For D_2_^15+^, a dendrimer with a mixed periphery, hydrophobic interactions of phenylene units with the hydrocarbon tails of lipids were observed, along with electrostatic complexation with liposomes. As a result, defects were formed in the bilayer, which led to irreversible interactions with lipid membranes wherein there was no migration of D_2_^15+^ between liposomes. A first-generation dendrimer, D_1_^6+^, which was characterized by small size, a high degree of hydrophobicity, and a rigid structure, when interacting with liposomes caused significant destruction of liposomal membranes. Evidently, this interaction was irreversible: the addition of salt did not lead to the dissociation of the complex.

## 1. Introduction

In the last decades, dendrimers have received attention in biomedicine due to their unique structure and performance [1,2,3,4]. Dendrimers have specific, three-dimensional structures and differ from linear polymers in their low viscosity, high solubility, and high reactivity. Their shape and volume can be easily controlled during synthesis, and their terminal functional groups can be easily modified. The peripheral branching framework of dendrimers provides graft sites for functional groups and bioactive molecules. Dendrimers have been suggested as vectors for gene delivery, as well as antibacterial or antiviral agents [5,6]. Recently, dendrimers were developed for drug delivery [7] since their bioactive molecules can be physically captured in the interior of a dendrimer or covalently bonded to surface groups. The above-mentioned applications require comprehensive analysis and characterization of dendrimer–cell interactions.

To predict the impact of water-soluble polymers on cell membranes and explore the physicochemical aspects of interactions of linear and dendritic polyelectrolytes with biomembranes, cell-mimetic objects such as spherical bilayer vesicles (liposomes) have been widely used [8,9]. Liposomes of various compositions and sizes are actively applied in model studies, aimed at developing new, effective systems with desired properties for biomedical use. Gradually expanding, such investigations nowadays cover a wide range of issues related to the efficiency of polymer adsorption on biomembranes of different compositions, the reversibility of polymer adsorption, the stability of the resulting complexes in water–salt media, structural rearrangements with biomembranes caused by polymer adsorption, migration of polymers between liposomes, and fusion and destruction of liposomes [10,11,12,13,14,15]. The results of model studies, together with the findings of cellular experiments, have made it possible to make significant progress in understanding the mechanisms of interaction between polymers and cells. It has been shown earlier that the binding of water-soluble polymers to cells can be followed by clustering of lipid molecules or membrane proteins, enhancement of the transmembrane migration of lipids, increase in membrane permeability toward inorganic ions, and incorporation of polyelectrolytes into the lipid bilayer [16,17,18,19,20,21]. It has also been established that the type of the interaction between polymers and the lipid bilayer and the properties of the resulting complexes are determined by the nature of the adsorbed polymer (the number of charged groups, the presence of hydrophobic fragments, etc.). The incorporation of hydrophobic groups, e.g., alkyl chains, in a polyelectrolyte’s structure also significantly affects adhesion on the membrane surface, the ability of penetration into the lipid bilayer, and the reversibility of polycation-to-liposome interaction [22]. The properties of a complex of liposomes with polybetaines are determined by the spacer length in the betaine group. Variation in the spacer makes it possible to control the behavior of polybetaine in contact with anionic liposomes, from the absence of any interaction to critical structural rearrangements caused by polymer adsorption [23]. The charge density of ionenes was shown to be a key factor that determined the method of their interaction with liposomes [24]. The interactions of dendrimers with the lipid bilayer have also been found to depend on the nature of a dendrimer’s functional groups: the nature of the binding of positively charged dendrimers with liposomes was found to be electrostatic, but interactions with neutral maltose-modified dendrimers occurred via hydrogen bonding [25,26]. It has been shown earlier that the mechanism of cytotoxic action of dendrimer-based systems strongly depends on dendrimer generation [27,28]. 

Previously, we showed the ability of cationic pyridylphenylene dendrimers (CPPDs) to disrupt amyloid protein aggregates [29]. CPPDs are amphiphilic macromolecules with hydrophilic positively charged pyridinium groups and hydrophobic phenylene groups localized in the inner part of the molecule. CPPDs interact with proteins and nucleic acids, forming stable complexes by means of electrostatic and hydrophobic interactions. It has been suggested that CPPDs can be used as nonviral transfection agents [30,31,32]. By means of an MTT assay, it was found that CPPDs demonstrated a moderate toxicity that decreases significantly upon complexation with protein [33]. In order to gain insight into the molecular mechanism of cell–dendrimer interactions and to clarify the mechanism of toxicity and the ability of cell penetration, we begin a detailed study of the interactions between cationic pyridylphenylene dendrimers and anionic liposomes. As a first step, we investigate and describe some peculiarities of interactions of a third-generation cationic pyridylphenylene dendrimer, D_3_^50+^, with multicomponent anionic liposomes [34]. It is found that the binding of the dendrimer to the membrane proceeds only through electrostatic forces, and D_3_^50+^ does not demonstrate a disruptive effect toward the lipid membrane. 

**Figure 1:**
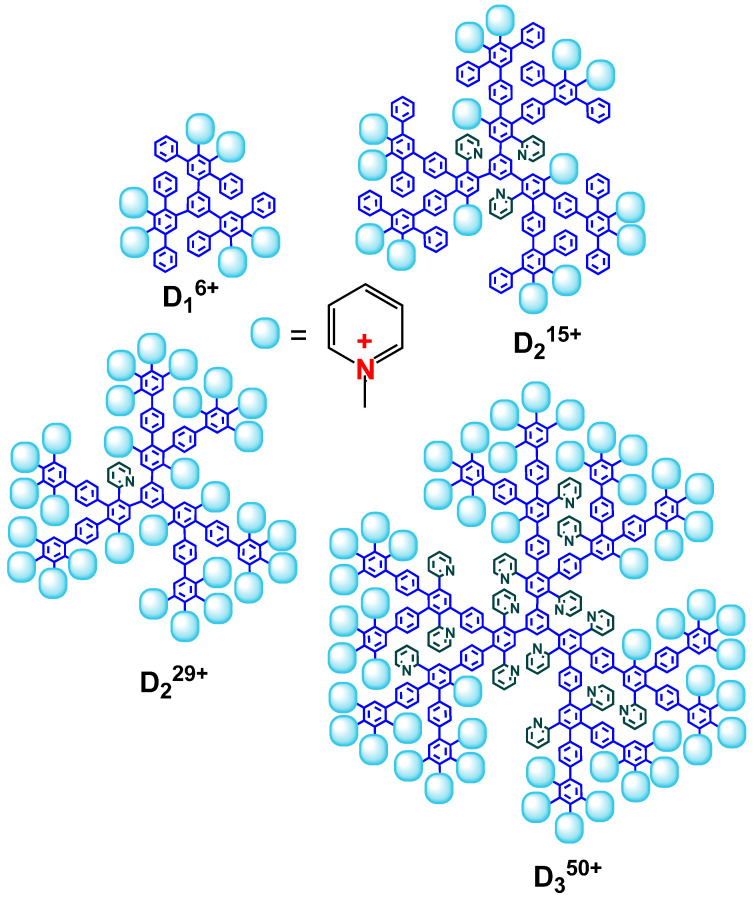
Structures of cationic pyridylphenylene dendrimers. Dendrimers have different hydrophobicity depending on the amount of charged pyridine groups. D_3_^50+^ is a third-generation dendrimer with a completely charged periphery, D_2_^29+^ and D_2_^15+^ are second-generation dendrimers characterized by completely charged (for D_2_^29+^) and mixed (for D_2_^15+^) periphery. A first-generation dendrimer with a mixed periphery, D_1_^6+^, is characterized by small size and a high degree of hydrophobicity.

Here, we report on the role of CPPD architecture, namely generation and the amounts of charged pyridynium groups and hydrophobic phenylene units, i.e., the degree of hydrophobicity and the rigidity of the spatial structure, on the method of interaction with small anionic liposomes. Liposomes are prepared from electroneutral dioleoylphosphatidylcholine (DOPC) and negatively charged cardiolipin (CL). Since cholesterol (Chol) is known to be an integral component of plasma membrane [35,36], it is added to the model lipid bilayer. We study the interactions of liposomes with CPPDs of the first, second, and third generation with mixed or completely pyridyl periphery (D_1_^6+^, D_2_^15+^, D_2_^29+^, and D_3_^50+^). We are particularly interested in finding the correlation between the structure of CPPDs (Figure 1) and the effect induced by dendrimers in the lipid membrane. We investigate the driving force of interaction, the ability of dendrimers to migrate between liposomes, the stability of dendrimer–liposome complexes in water–salt media, and possible disruptive effects toward the lipid bilayer. 

Such investigations may help to understand the mechanisms of action of dendrimers of different natures on cell membranes and to suggest the most optimal variant of dendrimer structure for biomedical applications.

## 2. Results and Discussion

Anionic CL/Chol/DOPC liposomes were characterized by means of laser microelectrophoresis and dynamic light scattering. The electrophoretic mobilities (EPMs) and hydrodynamic diameters of CL/Chol/DOPC liposomes of different concentrations are presented in Table 1. 

### 2.1. Complexes of Dendrimers with Anionic CL/Chol/DOPC liposomes

**Figure 2:**
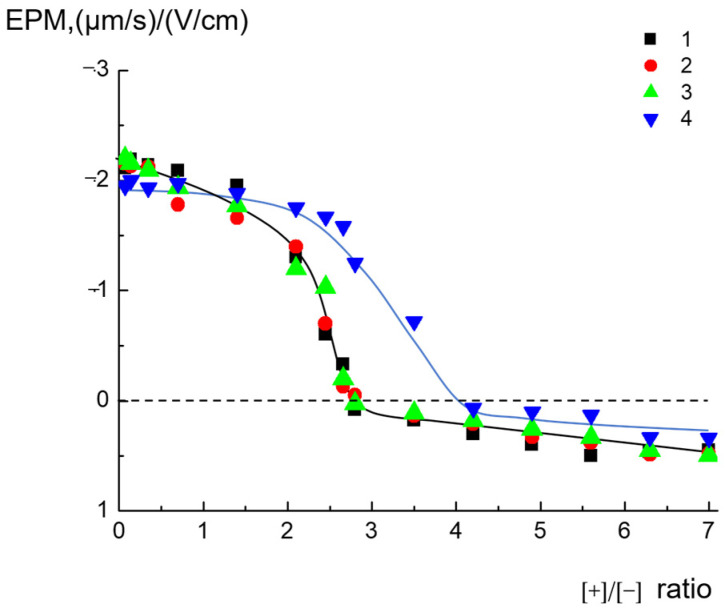
EPMs of the dendrimer–liposome complexes versus ratios of ionic groups [+]/[−] for D_3_^50+^ (1), D_2_^29+^ (2), D_2_^15+^ (3), and D_1_^6+^ (4), where [+] is the molar concentration of the pyridinium groups of dendrimers, and [−] is the molar concentration of negatively charged cardiolipin headgroups. CL/Chol/DOPC liposomes, [CL] = 1.5 × 10^−4^ mol/L, total lipid concentration of 1 mg/mL. 10^−3^ M phosphate buffer, pH 7.2.

The interactions of cationic pyridylphenylene dendrimers with anionic liposomes were studied via microelectrophoresis by registering the electrophoretic mobility associated with the surface charge of the particles. The obtained results are presented in Figure 2. As follows from the figure, the addition of all types of the dendrimers to anionic liposomes was accompanied by progressive liposome charge neutralization with further slight surface recharging (Figure 2). Hereafter, we use the ratio of the ionic groups [+]/[−], where [+] is the molar concentration of the pyridinium groups of a dendrimer, and [−] is the molar concentration of the negatively charged cardiolipin headgroups. The corresponding zeta potentials of the dendrimer - liposome complexes versus ratio of the ionic groups are presented at Figure S1, SI. A complete neutralization of the liposome charge was achieved at a molar ratio of [+]/[−] = 2.6 for D_2_^15+^, D_2_^29+^, and D_3_^50+^ (curves 1–3). This result is in agreement with our previous results for D_3_^50+^ and, apparently, was explained by the rigidity of the dendrimer structure. For D_3_^50+^, only a part of the pyridinium groups of the dendrimer molecules participated in electrostatic binding with liposomes. It was also found that D_3_^50+^ was unable to induce flip-flop in contrast to flexible polycations, which were capable of forming loops on the surfaces of liposomes [34]. It should be noted that, for D_1_^6+^, a complete neutralization of the liposome charge was achieved at a molar ratio of [+]/[−] = 4.2–4.6, that is, with an even greater amount of cationic groups (curve 4). One of the possible explanations for this effect may be the partial destruction of the structures of liposomes caused by D_1_^6+^. We return below in more detail to this hypothesis when describing further experiments.

**Figure 3:**
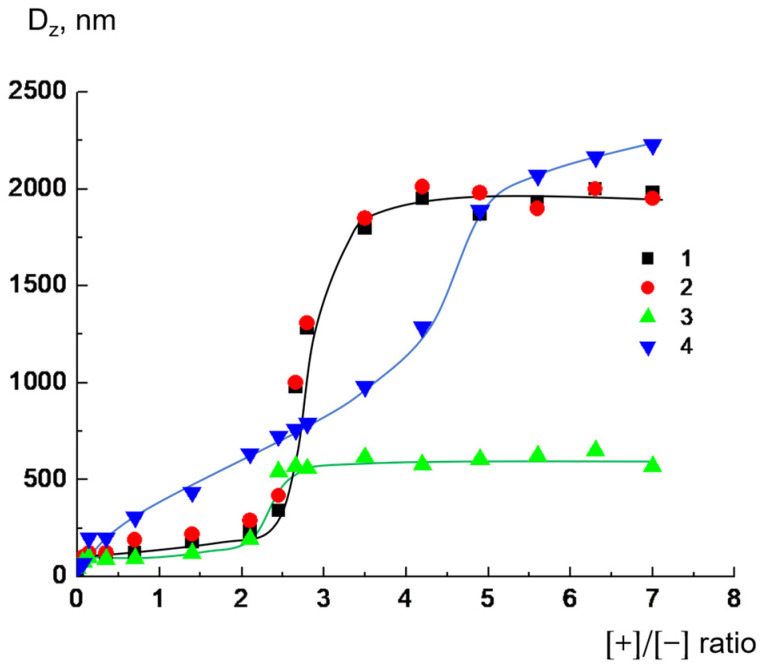
Hydrodynamic diameters of dendrimer–liposome complexes versus ratios of ionic groups [+]/[−] for D_3_^50+^ (1), D_2_^29+^ (2), D_2_^15+^ (3), and D_1_^6+^ (4), where [+] is the molar concentration of the pyridinium groups of dendrimers, and [−] is the molar concentration of negatively charged cardiolipin headgroups. CL/Chol/DOPC liposomes, [CL] = 1.5 × 10^−4^ mol/L, total lipid concentration of 1 mg/mL. 10^−3^ M phosphate buffer, pH 7.2.

In parallel, we examined how the mean hydrodynamic diameters of complex particles changed with increasing dendrimer concentration, namely the [+]/[−] ratio. The binding of dendrimers with liposomes was followed by enlargement in the particle size (Figure 3. The examples of the experimental autocorrelation curves for dendrimer-liposome complexes are given in Figure S2, SI). There was a correlation between the electrophoretic and light-scattering data; in all cases, the largest complex particles were formed when the liposome surface charge was completely neutralized by adsorbed dendrimer, that is, at EPM = 0 (Figure 2). For D_2_^15+^, D_2_^29+^, and D_3_^50+^, this was achieved at [+]/[−] = 2.6, and for D_1_^6+^, at [+]/[−] = 4.5. It should be noted that the curves for D_2_^29+^ and D_3_^50+^ practically coincided; the maximum size was about 2000 nm, while for D_2_^15+^, the maximum size was significantly smaller at ~500 nm. Obviously, the size of the complex was determined by the number of liposomes involved in the electrostatic interaction with the dendrimer, which, in turn, depended on how negatively charged cardiolipin molecules were distributed in the lipid bilayer. We assume that, in the case of D_2_^15+^, the number of liposomes involved in the formation of the aggregate was smaller, which led to a smaller maximum aggregate size. 

In all cases, at large excess of the dendrimer, no decrease in the particle size of the complex was observed. This was due minor surface recharging, which was not sufficient to prevent the aggregation process [34].

Thus, it was found that, for D_1_^6+^, the concentration required for complete neutralization of the liposome charge, as well as the formation of aggregates of maximum size, differed significantly from those of D_2_^15+^, D_2_^29+^, and D_3_^50+^. 

**Figure 4:**
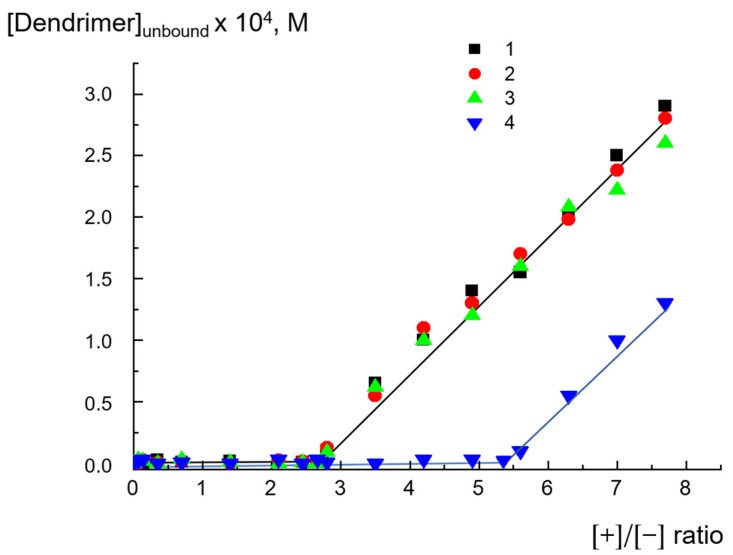
Dependence of concentration of unbound dendrimers on ratio of ionic groups [+]/[−] for D_3_^50+^ (1), D_2_^29+^ (2), D_2_^15+^ (3), and D_1_^6+^ (4), where [+] is the molar concentration of the pyridinium groups of dendrimers, and [−] is the molar concentration of negatively charged cardiolipin headgroups. CL/Chol/DOPC liposomes, [CL] = 1.5 × 10^−4^ mol/L, total lipid concentration of 1 mg/mL. 10^−3^ M phosphate buffer, pH 7.2.

We also observed similar behaviors of dendrimers by analyzing dependences on the degree of binding of D_1_^6+^, D_2_^15+^, D_2_^29+^, and D_3_^50+^ with liposomes. To determine whether all the amounts of dendrimer bound to anionic liposomes, the obtained dendrimer–liposome complexes were separated from the suspension by centrifugation (see details below), and then we analyzed the supernatant for the dendrimer concentration by means of UV spectroscopy. The dependences of dendrimer concentration in the supernatant on the ratios of the ionic groups [+]/[−] are presented in Figure 4. One can see that D_2_^15+^, D_2_^29+^, and D_3_^50+^ were completely bound with liposomes up to [+]/[−]~2.8, which corresponded to the concentration of dendrimers of ~2.3 × 10^−4^ mol/L. This result was in agreement with the EPM findings (Figure 2). The ratio slightly exceeded the value needed for complete neutralization of the liposome charge, thus explaining the low recharge of the liposomal surface and reaching a plateau after the neutralization point. D_1_^6+^ was completely bound with liposomes up to [+]/[−] = 5.4, which was also in accordance with the microelectrophoresis results but exceeded the values obtained for other dendrimers. Why did the behavior of D_1_^6+^ differ from that of D_2_^15+^, D_2_^29+^, and D_3_^50+^? For the next step, we checked if the integrity of liposomes was retained in contact with dendrimers or whether the adsorption of some was accompanied of defect formation in the lipid bilayer. This aspect of interaction is of key importance from the viewpoint of biomedical applications for dendrimers [19,37].

### 2.2. Integrity of Lipid Bilayer upon Complexation with Dendrimers

The integrity of liposomes complexed with dendrimers was controlled by conductometry (see Materials and Methods for details). Anionic CL/Chol/DOPC liposomes with 1M NaCl solution in the inner pool were complexed with cationic dendrimers. The ratio of the ionic groups [+]/[−] was equal to 2. For these ratios, the complexes were negatively charged, and dendrimers were completely bound with liposomes, but the sizes of the aggregates did not exceed 250–550 nm (Figure 2, Figure 3 and Figure 4).

**Figure 5:**
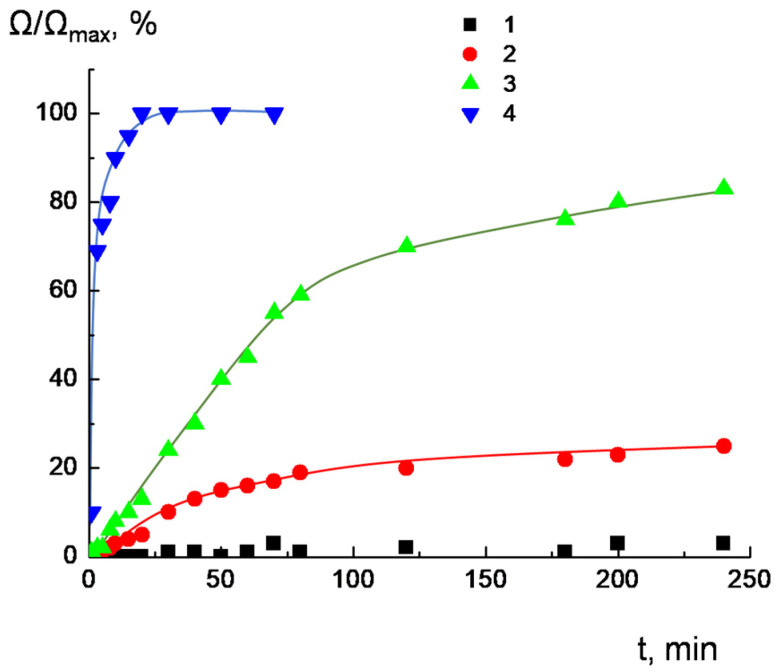
Time-dependent changes in relative conductivity of dendrimer–NaCl-loaded liposome complexes for D_3_^50+^ (1), D_2_^29+^ (2), D_2_^15+^ (3), and D_1_^6+^ (4). The ratio of the ionic groups was [+]/[−] = 2. CL/Chol/DOPC liposomes, [CL] = 1.5 × 10^−4^ mol/L, total lipid concentration of 1 mg/mL. 10^−3^ M phosphate buffer, pH 7.2.

A leakage of salt from the liposomes to the surrounding solution was monitored via measuring the suspension conductivity. The maximum increase in conductivity due to irreversible liposome destruction was achieved after the addition of a 10-fold excess of Triton X-100 surfactant. The difference in conductivity between the liposome suspension filled with NaCl and the liposome suspension completely destroyed by Triton X-100 was taken as a unit of relative conductivity. The appearance of defects in the membranes of anionic liposomes should be accompanied by a leakage of salt from liposomes into the surrounding solution and an increase in the electrical conductivity of the suspension. The results of the experiment are presented in Figure 5. Liposomes responded to complexation with dendrimers in different manners. Decreases in dendrimer generation and the number of charged groups, as well as an increase in the degree of hydrophobicity, resulted in accelerating NaCl leakage. No change in the conductivity was observed for D_3_^50+^ within 250 min after complexation with liposomes (curve 1), which definitely indicated the preservation of liposome integrity in the resulting complex. Complexation of liposomes with D_2_^29+^ was followed by minor leakage of salt into the surrounding solution; in the first 30 min, only 3–5% of the NaCl was released, and in 250 min, ≤20% of the NaCl was released. For D_2_^15+^, the effect became more pronounced: in the first 30 min, 25% of the NaCl was released, while 80% of the NaCl was released within 250 min after complex formation. Finally, for D_1_^6+^, 70–80% of the NaCl flowed out within the first minutes after complexation with liposomes, and in 30 min, the salt was completely released from the internal cavities of the liposomes.

**Figure 6:**
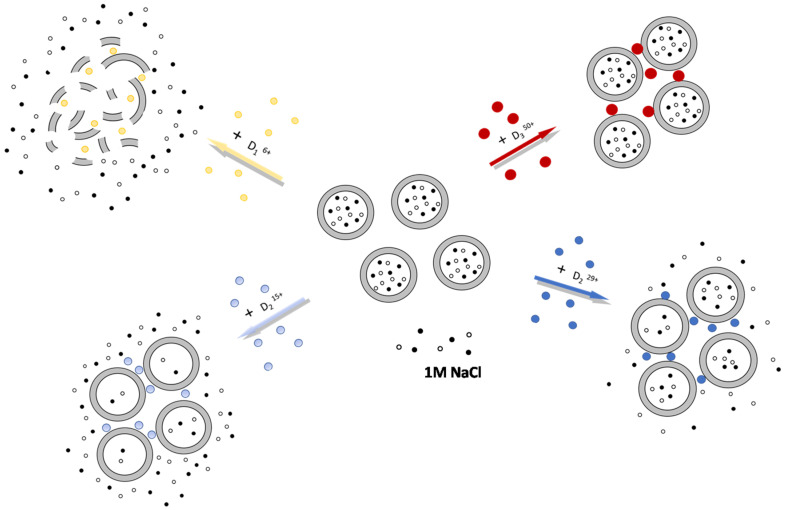
Schematic representation of interactions of liposomes with dendrimers. D_3_^50+^ and D_2_^29+^ did not display a disruptive effect toward the liposomes, while the complexation of liposomes with D_2_^29+^ was followed by minor defect formation. D_1_^6+^ induced significant destruction of liposomal membranes.

This behavior of the complexes containing D_2_^15+^ and D_2_^29+^ could be explained by the partial disturbance of the integrity of the liposomal membrane upon dendrimer adsorption. D_1_^6+^ apparently induced significant defect formation and a complete release of the NaCl (Figure 6).

Previously, for linear polycations, it has been shown that such formation of defects in the lipid bilayer is usually accompanied by irreversible interaction of the polymer with the membrane. The polymer may embed into the bilayer via the sites of defects in the packing of the lipid bilayer. If this is so, the formation of complexes becomes irreversible. We tested this assumption for dendrimer–liposome complexes using fluorescence spectroscopy.

### 2.3. Reversibility of Interactions 

**Figure 7:**
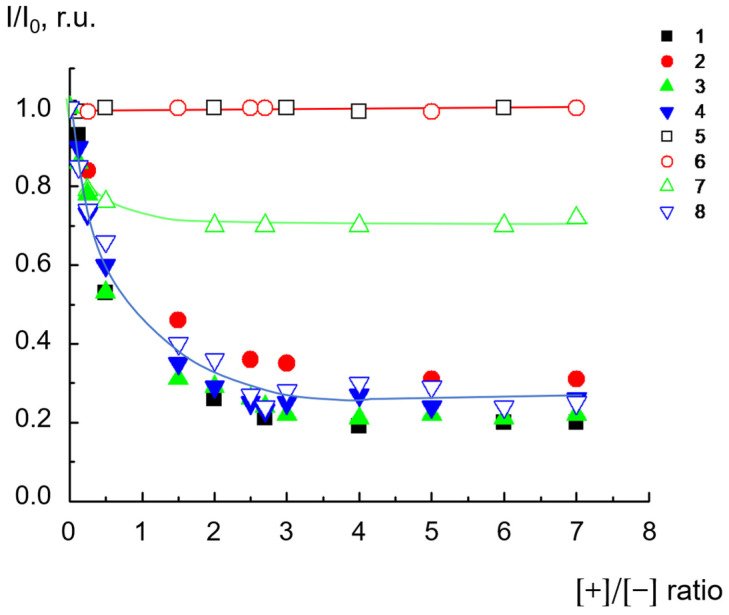
Relative fluorescence intensity of label (DOPE-CF) in dendrimer–liposome complexes as a function of ratios of ionic groups [+]/[−] for D_3_^50+^ (1, 5), D_2_^29+^ (2, 6), D_2_^15+^ (3, 7), and D_1_^6+^ (4, 8) before (1–4) and after (5–8) NaCl addition. [NaCl] = 0.4 mol/L. CL/Chol/DOPC liposomes, [CL] = 1.5 × 10^−4^ mol/L, total lipid concentration of 1 mg/mL. 10^−3^ M phosphate buffer, pH 7.2.

Since cationic polymers containing pyridinium groups are typical fluorescence quenchers, reversibility of their complexation with liposomes can be monitored by means of fluorescence spectroscopy in the fluorescence intensity of the label incorporated into the lipid bilayer [18]. The experiment was performed with the same concentrations used for the conductometric investigations. As follows from the data in Figure 7 (curves 1–4), for all types of dendrimers, the fluorescence intensity of the label was decreased due to adsorption on the liposomal surface. Then, low-molecular-weight electrolyte NaCl was added to the complexes at a concentration of 0.4 mol/L. 

For D_3_^50+^ and D_2_^29+^, complete fluorescence recovery upon the addition of NaCl to the complexes was observed (curves 5 and 6). In this case, an increase in the ionic strength, namely, the addition of salt, was followed by the destruction of the electrostatic contacts of the dendrimer–liposome complexes. The process was completed within 10 s, revealing the absolute removal of dendrimers from the liposomal surface. The removal of D_3_^50+^ and D_2_^29+^ was accompanied by the disaggregation of complex particles and a decrease in their size from 250 nm down to 40 ± 8 nm, which was equal to the size of the initial liposomes. 

For D_2_^15+^, recovery of fluorescence intensity up to the level of 0.7 was observed, thus indicating partial dissociation of the complex fluorescence (Figure 7, curve 7). In this case, the particle size was not completely restored: the hydrodynamic diameter of the particles decreased from 250 nm to 150 nm, which exceeded the size of the original liposomes. This means that salt was not able to completely remove D_2_^15+^ from the surfaces of the liposomes.

For D_1_^6+^, the addition of salt caused no rise in the intensity of fluorescence (Figure 7, curve 8). In this case, there was practically no change in particle size in the system at all: it remained at the level of 500-550 nm. This result definitely indicates that the D_1_^6+^–liposome complex did not dissociate into the initial components under the increase in ionic strength. 

These findings correlated well with the results of the investigation of liposome integrity in complexes with dendrimers. The interaction of third-generation cationic dendrimer D_3_^50+^, with an almost fully charged periphery, with anionic liposomes was electrostatic in nature. In the first hours, no significant defects were generated in the lipid bilayer under the action of D_3_^50+^. As a result, the interaction was reversible: the dendrimers could be completely removed from the surfaces of liposomes under the increase in ionic strength upon salt addition. The nature of the interaction of the second-generation dendrimer with liposomes was determined by the numbers of charged pyridynium groups and hydrophobic phenylene units, i.e., the degree of hydrophobicity. D_2_^29+^, with an almost fully charged periphery, reversibly interacted with liposomes, despite the formation of a small number of defects. It could be detached from the liposome complex by adding NaCl. The fluorescence experiments found that D_2_^15+^ did not leave the liposomal interface when NaCl was added in an amount that was sufficient for the destruction of salt bonds responsible for electrostatic adsorption. As has been shown earlier, hydrophobic “anchors” in macromolecules stabilize their complexes with liposomes due to the incorporation of hydrophobic parts into the lipid bilayer [22,37]. In the case of D_2_^15+^, a dendrimer with a mixed periphery that contained both hydrophobic phenylene units and cationic groups, a similar situation could be observed. In this case, the nature of the interaction appeared to be mixed: in addition to electrostatic contacts between oppositely charged cationic groups of the dendrimer and anionic cardiolipin molecules, hydrophobic interactions of phenylene units with the hydrocarbon tails of lipids took place. Such an interaction could be accompanied by the formation of defects in the membrane, which was confirmed by conductometry. These data were also consistent with the results of the size measurements: NaCl injection into suspensions of dendrimer–liposome complexes caused only a partial decrease in particle size. Even with excess salt, the size of the particles exceeded the size of the initial liposomes. Finally, the first-generation dendrimer of D_1_^6+^, characterized by small size, a high degree of hydrophobicity, and a rigid structure, when interacting with liposomes, caused defect formation apparently followed by the significant destruction of liposomal membranes. Obviously, in this case, there was no restoration of fluorescence intensity since the liposomes were destroyed and new structures were formed in which the label continued to be in direct contact with the dendrimer. This was also confirmed by the results of dynamic light-scattering (DLS) experiments. As mentioned above, the sizes of the complexes remained unchanged after NaCl addition. This suggestion explained the differences in the behavior of D_1_^6+^ and the other investigated dendrimers upon interaction with liposomes. We previously found that, for D_1_^6+^, the concentration required for complete neutralization of the liposome charge (Figure 2), as well as the formation of aggregates of maximum size (Figure 3), differed significantly from those for D_2_^15+^, D_2_^29+^, and D_3_^50+^. The experiments on the degree of binding of dendrimers with liposomes also revealed a difference: the results for D_1_^6+^ were in accordance with the microelectrophoresis and DLS results, but the values were higher than those obtained for other dendrimers. The assumption of liposomal destruction upon interaction with D_1_^6+^ explained this distinction. As a result of the destruction, all the anionic cardiolipin molecules, even those that were located at the inner leaflet of the membrane, were involved in the interaction with the cationic groups of D_1_^6+^. This led to an increase in the concentration of dendrimer needed for complete neutralization of the negative charge.

### 2.4. Migration of Dendrimers between Liposomes 

Usually, before fixing onto a target cell surface polyelectrolyte-based drug formulations electrostatically interact with random cells in biological fluid [38]. In this regard, it is very important that the process of polymer interaction with nontarget cells be reversible. The ability of polyelectrolytes to migrate among nanosized particles has been demonstrated in experiments with colloid particles of different morphologies: monodispersed latex particles and liposomes [39,40]. Therefore, we decided to check if the dendrimer was able to migrate from one liposome to another. The experiments were carried out for dendrimers that did not destroy liposomes during interaction, namely, for D_3_^50+^, D_2_^29+^, and D_2_^15+^. 

The ability of the adsorbed dendrimers to migrate between CL/Chol/DOPC liposomes was estimated by fluorescence spectroscopy. The details of the experiment are described below in Section 3. We tested two types of complexes with different ratios of ionic groups, but for these ratios, the complexes were negatively charged, the dendrimers were completely bound with liposomes, and the sizes of the aggregates did not exceed 250 nm. For sample 1, at the minimal dendrimer concentration, the particle size was comparable with the size of individual liposomes, indicating that the complex consisted of individual liposomes with dendrimer molecules adsorbed on their surfaces. In this case, the dendrimer migration between individual liposomes was studied. For sample 2, slight aggregation was observed and, in this case, several liposomes were involved in complex formation.

If a dendrimer was capable of migrating between liposomes, its movement from fluorescently labeled liposomes (Lip*) to unlabeled ones (Lip) was accompanied by fluorescence recovery. During 1.5 h, the relative fluorescence intensity was investigated, and the concentrations of dendrimers bound with labeled CL/Chol/DOPC liposomes were determined with calibration curves. The difference between the dendrimer concentration used for complexation and the concentration calculated from the calibration curve corresponded to the amount of dendrimers that migrated to unlabeled liposomes added to the suspension.

**Figure 8:**
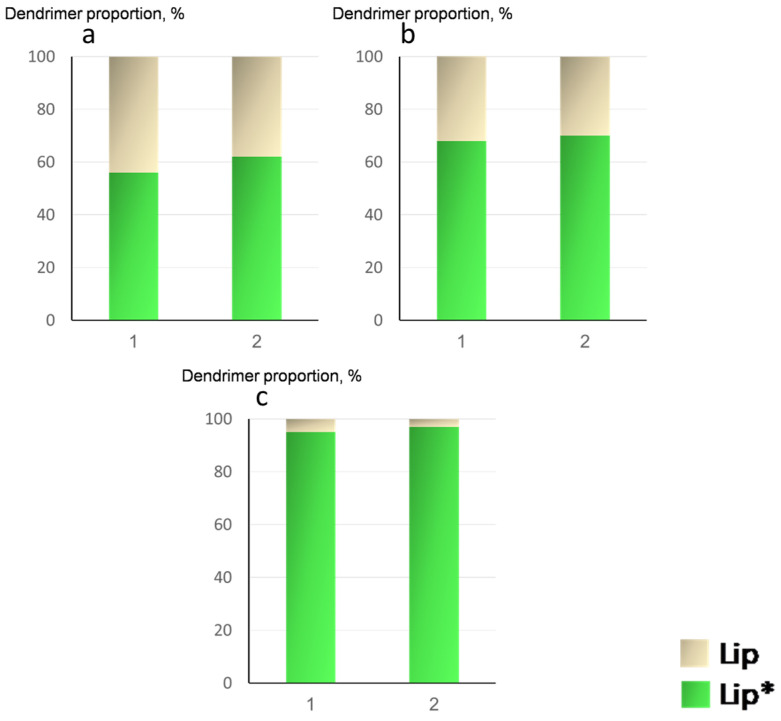
Diagrams of distribution of dendrimers between labeled (Lip*) and unlabeled (Lip) liposomes in 1.5 h for D_3_^50+^ (**a**), D_2_^29+^ (**b**), and D_2_^15+^ (**c**). The ratios of the ionic groups were [+]/[−] = 0.5 (1) and 1 (2). CL/Chol/DOPC liposomes, [CL] = 1.5 × 10^−4^ mol/L, total lipid concentration of 1 mg/mL. 10^−3^ M phosphate buffer, pH 7.2.

From the concentration relation, we established a dendrimer distribution between labeled (Lip*) and unlabeled (Lip) liposomes. The results of the experiments, clearly indicating the ability of the dendrimers to migrate between liposomes, are presented in Figure 8. It follows from Figure 8 that, in 1.5 h, the distribution of D_3_^50+^ was close to uniform (diagram A). For D_2_^29+^, the migration was not so effective (diagram B): about 30–35% of the dendrimer molecules were transferred to unlabeled liposomes from labeled ones. D_2_^15+^ practically did not migrate between liposomes: in 1.5 h, only 3–5% of the dendrimer molecules were transferred to unlabeled liposomes from labeled ones. This means that the migration process depended on the dendrimer structure.

**Figure 9:**
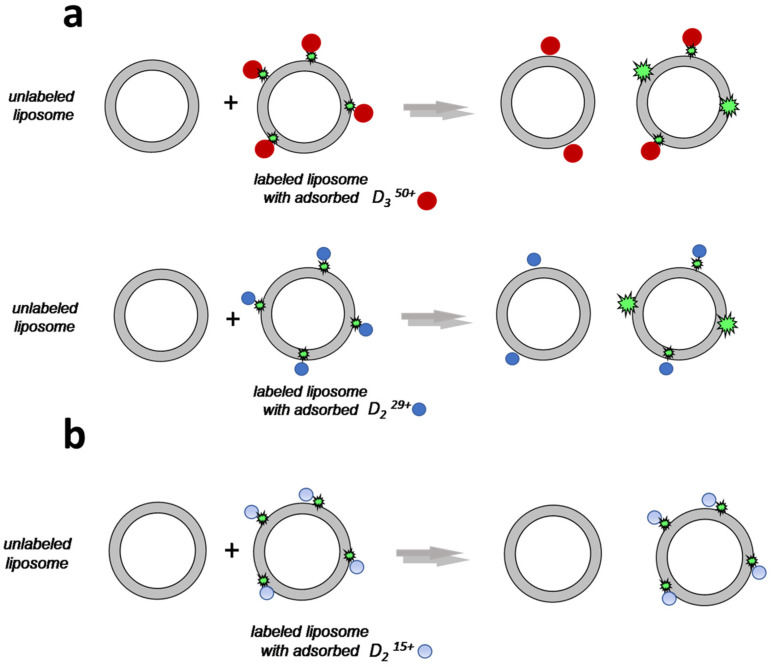
Schematic representation of the migration experiment for D_3_^50+^ and D_2_^29+^ (**a**), as well as for D_2_^15+^ (**b**). D_3_^50+^ and D_2_^29+^ were able to migrate between liposomes since the binding was due to electrostatic forces (**a**). For D_2_^15+^, hydrophobic interactions were observed, along with electrostatic complexation; as a result, defects were formed in the membrane that led to irreversible interactions with no migration of D_2_^15+^ between liposomes (**b**). The dendrimer molecules are indicated as circles, and the fluorescent labels are indicated as green stars.

As we discussed above, the interactions of third- and second-generation dendrimers with almost fully charged periphery (D_3_^50+^ and D_2_^29+^) with anionic liposomes were electrostatic in nature. Practically no defects were formed upon dendrimer complexation with liposomes. Such dendrimers were able to migrate between liposomes (Figure 9a). In the case of D_2_^15+^, through complexation with liposomes hydrophobic interactions of phenylene units with the hydrocarbon tails of lipids presumably took place, along with electrostatic interactions. As a result, defects were formed in the membrane, which led to irreversible interactions of dendrimers with liposomes. This appeared to result in the dendrimers being unable to migrate between liposomes (Figure 9b).

## 3. Materials and Methods

### 3.1. Dendrimer Synthesis and Characterization 

Dendrimer synthesis was described previously [32]. Briefly, a hydrophobic parent pyridylphenylene dendrimer was synthesized through Diels Alder cycloaddition and desilylation reactions at the first stage. Next, alkylation of the pyridine groups with dimethylsulfate resulted in water-soluble products. The alkylation degree that was equal to the dendrimer charge was derived from an elemental analysis of sulfur content. It is important to note that dendrimers possess a permanent charge independent from pH range since it arises from quaternary pyridines. The size of the compounds was determined by dynamic light scattering. The dendrimer characteristics are summarized in Table 2. The structures of the dendrimers are presented in Figure 1.

### 3.2. Chemicals

Diphosphatidyl glycerol (cardiolipin, CL), 1,2-dioleoyl-sn-glycero-3-phosphocholine (DOPC), cholesterol (Chol), and 1,2-dioleoyl-sn-glycero-3-phosphoethanolamine-N-(carboxyfluorescein) (ammonium salt, DOPE-CF) (all from Avanti polar lipids, Alabaster, AL, USA) were used as received (the structures are presented in Table 3). Disodium hydrogen phosphate dihydrate (Na_2_HPO_4_⋅2H_2_O), sodium dihydrogen phosphate dihydrate (NaH_2_PO_4_⋅2H_2_O), and sodium chloride (all chemically pure grade and were purchased from Sigma-Aldrich, Darmstadt, Germany) were used as received. 10^–3^ M buffer solution was prepared by weighing. 

Double-distilled water was used for making solutions after additional treatment with a Milli-Q (Merck Millipore, Darmstadt, Germany) system that included ion exchange adsorption columns for advanced purification from organic impurities and filters for removing large particles. The conductivity of the purified water was 0.5 μS/cm.

### 3.3. Synthesis of Anionic Liposomes

Small unilamellar CL/Chol/DOPC liposomes were prepared via mixing methanol–chloroform (1:1 *wt*:*wt*) solutions of lipids, evaporating the organic solvent under a vacuum at 40 °C, and dispersing the lipid film in 10^−2^ M phosphate buffer solution (pH = 7.2), followed by final sonification [14] of the resulting lipid and water mixture with a Cole-Parmer 4710 ultrasonic homogenizer (Vernon Hills, IL, USA) for 400 s (2 × 200 s) at 20 °C. The resulting liposomes were separated from titanium dust via centrifugation in a J-11 centrifuge (Beckman, Brea, CA, USA) for 5 min at 11,000 rpm. The molar fraction of negatively charged CL headgroups was ν_CL_ = 2[CL]/(2[CL] + [DOPC]) = 0.1 (each CL molecule had two anionic groups). The mass fraction of cholesterol was equal to 0.1. 

Liposomes loaded with 1 M NaCl were prepared via dispersion of the lipid film in 10^−3^ M phosphate buffer solution (pH = 7.2) containing 1 M NaCl, followed by 2 h of dialysis of the suspension against 10^−3^ M buffer, which was renewed every 30 min.

Liposomes with fluorescent labels embedded in the bilayer were obtained by adding 1,2-dioleoyl-sn-glycero-3-phosphoethanolamine-N-(carboxyfluorescein, DOPE-CF) (ammonium salt) (from Avanti polar lipids, Alabaster, AL, USA) (0.1 wt % of total amount of lipids) to the mixtures of lipid solutions. Further procedures were similar to those described above.

The sizes of liposomes fluctuated from sample to sample but always remained within a 40 ± 10 nm range. A suspension of 2 mg/mL liposomes was progressively diluted with buffer solution to 0.2 mg/mL; meanwhile the size and surface charge (EPM) of liposomes were measured. The electrophoretic mobilities (EPMs) and hydrodynamic diameters of CL/Chol/DOPC liposomes of different concentrations are presented in Table 1. As follows from the data obtained, the sizes and EPMs of liposomes did not change significantly upon dilution.

### 3.4. Preparation of Dendrimer–Liposome Complexes 

Dendrimer–liposome complexes of 1 mL volume were obtained by the sequential addition of components. First, 100 μL of liposome suspension with a concentration of 10 mg/mL was diluted with (1000 − x) μL 10^–3^ M phosphate buffer (pH = 7.2), where x was the volume of 10^−3^ M aqueous solution of the dendrimer. Then, x μL of the dendrimer was added. Thus, the concentration of liposomes was constant (1 mg/mL), while the concentration of the dendrimer varied. Measurements were carried out within 5 min after addition of all the components. 

### 3.5. Degree of Binding of Dendrimers with Anionic Liposomes 

To determine the degree of binding of dendrimers with anionic liposomes, the following experiment was carried out. The dendrimer–liposome complexes were separated from the solution by centrifugation with a Beckman J2-21 centrifuge (Brea, CA, USA) for 40 min at 18,000 rpm. The dendrimer concentration in the supernatant was determined by UV spectroscopy using a UV-Mini spectrophotometer (Shimadzu, Kyoto, Japan) at the characteristic absorption band of λ = 257 nm.

### 3.6. Dendrimer Migration Experiment 

The possibility of dendrimer migration was investigated by means of fluorescence spectroscopy. The experiment was carried out for complexes with ratios of cationic groups of dendrimers to anionic CL groups [+]/[−] equal to 0.5 (sample 1) and 1 (sample 2). For these ratios, complexes were negatively charged and dendrimers were completely bound with liposomes, but the size of the aggregates did not exceed 250 nm. Firstly, complexes of dendrimers with fluorescently labeled CL/Chol/DOPC* liposomes were formed, followed by fluorescence quenching of labels embedded in the bilayer. Thus, a calibration curve (exponential dependence of relative fluorescence intensity I/I_0_ on dendrimer concentration) was obtained. Then, the test samples were formed: unlabeled CL/Chol/DOPC liposomes were added to dendrimer–CL/Chol/DOPC* liposome complexes. After 1.5 h, the relative fluorescence intensity was measured, and the concentration of dendrimers bound with labeled CL/Chol/DOPC* liposomes was determined with a calibration curve. 

### 3.7. Methods

Mean hydrodynamic diameters of liposomes and dendrimer–liposome complexes were measured using dynamic light scattering (DLS) at a fixed scattering angle (90°) in a thermostatic cell with a Brookhaven Zeta Plus instrument (Holtsville, NY, USA). Software provided by the manufacturer was employed to calculate diameter values (error ±7%). 

Electrophoretic mobility (EPM) of liposomes was determined in a thermostatic cell by laser microelectrophoresis with a Brookhaven Zeta Plus instrument (Holtsville, NY, USA) and corresponding software (error ±3–5%). 

The pH values of solutions were measured with a Radiometer pHM 83 pH meter (Copenhagen, Denmark) equipped with a measuring P1041 glass electrode and a K4041 calomel reference electrode (error ±0.02 units).

The conductivities of solutions were determined with a Radiometer CDM 83 conductometer (Copenhagen, Denmark) equipped with a PP1042 platinum electrode (error ±0.01 units). 

Fluorescence intensities of suspensions were determined using a F-4000 spectrofluorometer (Hitachi, Tokyo, Japan). Measurements were carried out in 1 cm quartz cuvettes (error ±5) in the quantitative mode at excitation and emission wavelengths of λex = 495 nm and λem = 526 nm, respectively. Note that there was no dendrimer absorption at these wavelengths.

Measurement of the sedimentation of dendrimer–liposome complexes was carried out with Beckman J2-21 centrifuge (Brea, CA, USA) for 40 min at 18,000 rpm. The dendrimer concentration in the supernatant was determined by UV spectroscopy using a UV-Mini spectrophotometer (Shimadzu, Kyoto, Japan) at the characteristic absorption band of λ = 257 nm.

All experiments were carried out in quadruplicate.

## 4. Conclusions

The results obtained enabled us to describe the behavior of cationic pyridylphenylene dendrimers of first, second, and third generation with mixed or completely pyridyl periphery (D_1_^6+^, D_2_^15+^, D_2_^29+^, and D_3_^50+^), in contact with cholesterol-containing (CL/Chol/DOPC) anionic liposomes as follows. All the dendrimers interacted with CL/Chol/DOPC liposomes, wherein the interactions were accompanied by liposome charge neutralization, particle size increase, and fluorescence quenching of labels embedded in the liposome bilayer. The architecture of the dendrimer, namely the generation, amount of charged pyridynium groups, amount of hydrophobic phenylene units, and rigidity of spatial structure, determined the special features of the dendrimer–liposome interactions. Dendrimers of the third and second generation with almost fully charged periphery (D_3_^50+^ and D_2_^29+^) did not display a disruptive effect toward the liposomes and did not penetrate into the hydrophobic lipid bilayer. Practically no defects were formed upon dendrimer complexation with liposomes. The binding of these dendrimers to the membrane surfaces was due to electrostatic forces: the dendrimer molecules could be removed from the liposome surfaces by low-molecular-weight electrolyte addition. Such dendrimers were able to migrate between liposomes. For D_2_^15+^, a dendrimer with a mixed periphery, hydrophobic interactions of phenylene units with the hydrocarbon tails of lipids were observed, as well as electrostatic complexation with liposomes. As a result, defects were formed in the membrane, which led to irreversible interactions of dendrimers with liposomes, wherein there was no migration of D_2_^15+^ between liposomes. The first-generation dendrimer of D_1_^6+^, characterized by small size, a high degree of hydrophobicity, and a rigid structure, when interacting with liposomes caused significant destruction of liposomal membranes. Evidently, this interaction was irreversible: the addition of salt did not lead to the dissociation of the complex. The obtained results are of interest for considering the possible biomedical applications of pyridylphenylene dendrimers.

## Figures and Tables

**Table 1:** EPMs and particle sizes (hydrodynamic diameter) of anionic CL/Chol/DOPC liposomes of different concentrations.

	Total Lipid Concentration, mg/mL					
	2	1	0.8	0.6	0.4	0.2
EPM, (μm/s)/(V/cm)	−2.35 ± 0.14	−2.45 ± 0.15	−2.38 ± 0.10	−2.41 ± 0.13	−2.42 ± 0.12	−2.43 ± 0.09
Hydrodynamic diameter, nm	45 ± 5	42 ± 4	39 ± 2	44 ± 2	34 ± 4	43 ± 7

**Table 2:** Characterization of pyridylphenylene dendrimers.

Dendrimer	Alkylation Degree, %	Number of Ionogenic Groups	Molecular Weight, Da	Hydrodynamic Diameter, nm
D_1_^6+^	100	6	1981	3.0
D_2_^15+^	83	15	5142	5.0
D_2_^29+^	96	29	7190	4.5
D_3_^50+^	78	50	14,440	5

**Table 3:** Lipids for liposome preparation.

Lipid	Formula	Molar Content in Liposomes
CL	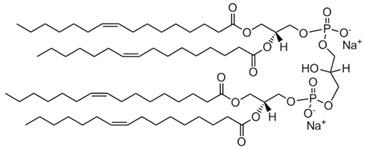	0.1 *
DOPC	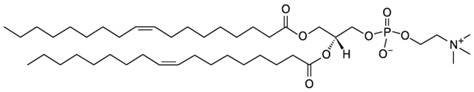	0.9
Chol	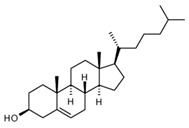	0.1
DOPE-CF	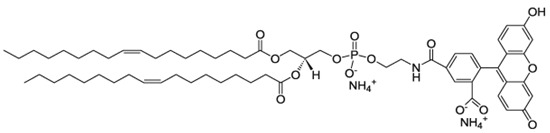	0.001

* Molar content of anionic CL headgroups of νCL = 2[CL]/(2[CL] + [DOPC]).

## Data Availability

The data are available from the corresponding author upon request.

## Supplementary Materials

The following supporting information can be downloaded at: https://www.mdpi.com/article/10.3390/ijms24032225/s1. Figure S1: Zeta potential of the dendrimer-liposome complexes versus ratio of the ionic groups [+]/[−] for D_3_^50+^(1), D_2_^29+^(2), D_2_^15+^(3) and D_1_^6+^(4), where [+] is the molar concentration of pyridinium groups of the dendrimer and [−] is the molar concentration of negatively charged cardiolipin headgroups. CL/Chol/DOPC liposomes, [CL] = 1.5 × 10^−4^ mol/L, total lipid concentration 1 mg/mL. 10^−3^ M phosphate buffer, pH 7.2. Figure S2: Examples of the experimental autocorrelation curves for dendrimer-liposome complexes for D_3_^50+^(1), D_2_^29+^(2), D_2_^15+^(3) and D_1_^6+^(4) with different ratio of the ionic groups [+]/[−], where [+] is the molar concentration of pyridinium groups of the dendrimer and [−] is the molar concentration of negatively charged cardiolipin headgroups. [+]/[−]= 2.5 (1), 2.7 (2), 0.1 (3), 1.4 (4). CL/Chol/DOPC liposomes, [CL] = 1.5 × 10^−4^ mol/L, total lipid concentration 1 mg/mL. 10^−3^ M phosphate buffer, pH 7.2.
