# Supplementary material for: Complexes of Cationic Pyridylphenylene Dendrimers with Anionic Liposomes: The Role of Dendrimer Composition in Membrane Structural Changes
Source: Int J Mol Sci. 2023 Jan 22;24(3):2225. doi: 10.3390/ijms24032225 (PMC9917332; doi:10.3390/ijms24032225)
Supplement: Supplementary file 1 [file ijms-24-02225-s001.zip › ijms-2149283-supplementary.pdf]

Complexes of Cationic Pyridylphenylene Dendrimers with Anionic Liposomes: The Role of Dendrimer’s Composition in Membrane Structural Changes

Anna A. Efimova [1], Svetlana A. Sorokina [2], Kseniya S. Trosheva [1], Alexander A. Yaroslavov [1] and ZinaidaB. Shifrina [2,*]

1. Department of Chemistry, M.V. Lomonosov Moscow State University, 1-3 Leninskie Gory, 119991 Moscow, Russia
2. A.N. Nesmeyanov Institute of Organoelement Compounds, Russian Academy of Sciences, 28 Vavilov St., 119991 Moscow, Russia

* Correspondence: shifrina@ineos.ac.ru

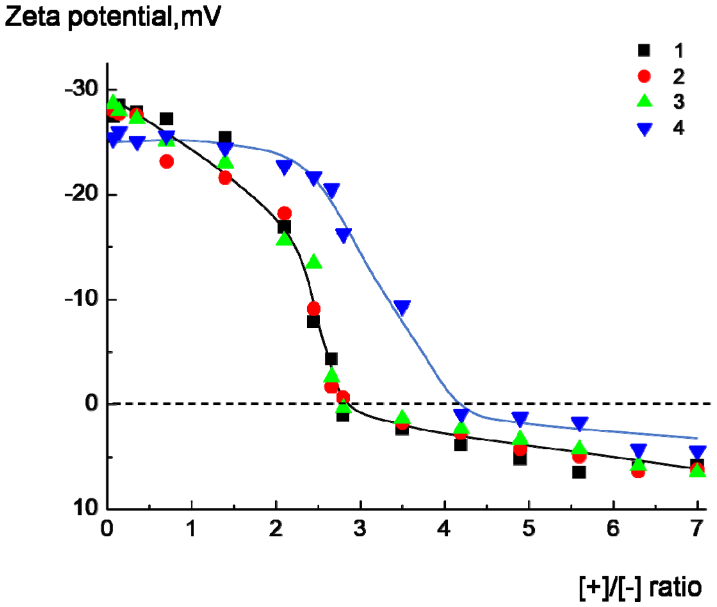

Figure S1. Zeta potential of the dendrimer - liposome complexes versus ratio of the ionic groups [+]/[-]for D_3^{50+}(1),D_2^{29+}(2), D_2^{15+}(3) and D_1^{6+}(4), where [+] is the molar concentration of pyridinium groups of the dendrimer and [-] is the molar concentration of negatively charged cardiolipin headgroups. CL/Chol/DOPCliposomes, [CL] = 1.5 × 10^{-4} mol/L, total lipid concentration 1 mg/mL. 10^{-3} M phosphate buffer, pH 7.2.

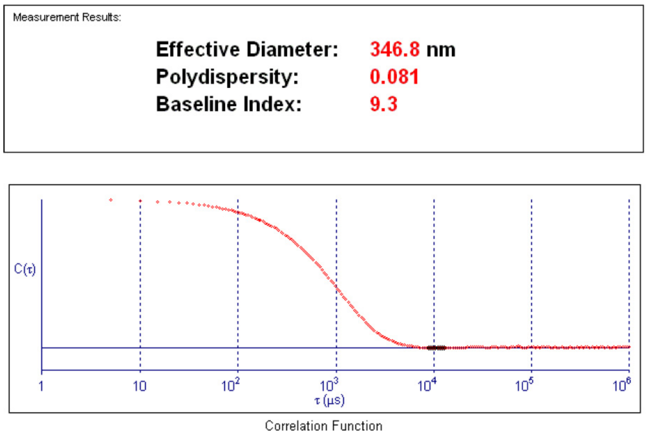
Measurement Results:
Effective Diameter: 346.8 nm
Polydispersity: 0.081
Baseline Index: 9.3
C(τ)
1 10 10^2 10^3 10^4 10^5 10^6
τ (μs)
Correlation Function

(1)

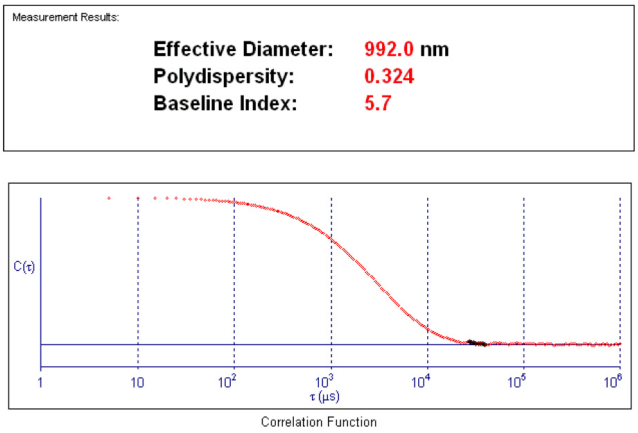
Measurement Results:
Effective Diameter: 992.0 nm
Polydispersity: 0.324
Baseline Index: 5.7
C(τ)
1 10 10^2 10^3 10^4 10^5 10^6
τ (μs)
Correlation Function

(2)

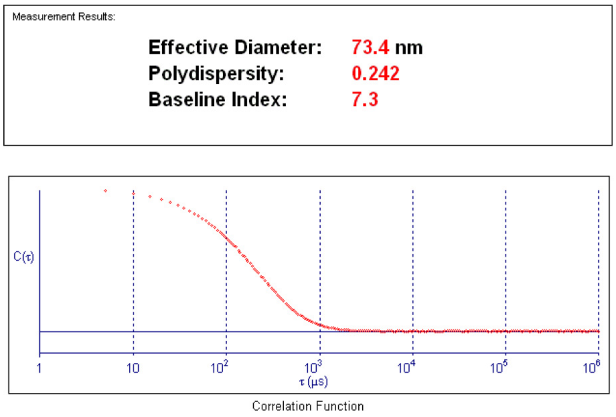
Measurement Results:
Effective Diameter: 73.4 nm
Polydispersity: 0.242
Baseline Index: 7.3
C(τ)
1 10 10^2 10^3 10^4 10^5 10^6
τ (μs)
Correlation Function

(3)

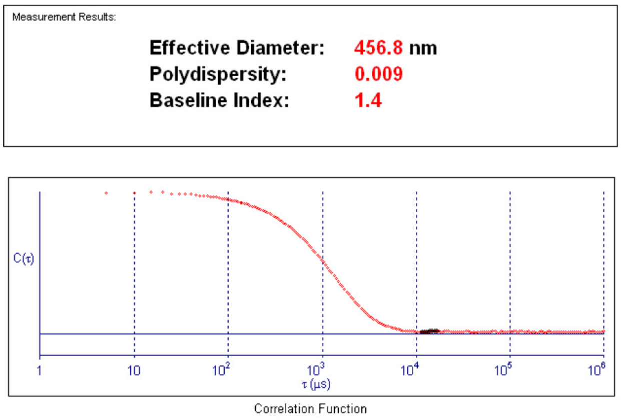

(4)

Figure S2. Examples of the experimental autocorrelation curves for dendrimer- liposome complexes for D_3^{50+}(1),D_2^{29+}(2), D_2^{15+}(3) and D_1^{6+}(4) with different ratio of the ionic groups [+]/[-], where [+] is the molar concentration of pyridinium groups of the dendrimer and [-] is the molar concentration of negatively charged cardiolipin headgroups. [+]/[-]= 2.5 (1), 2.7 (2), 0.1 (3), 1.4 (4). CL/Chol/DOPC liposomes, [CL] = 1.5×10^{-4} mol/L, total lipid concentration 1 mg/mL. 10^{-3} M phosphate buffer, pH 7.2.
